# Auditory and Visual Hallucinations Associated With Nitrofurantoin Use in an Inpatient Setting: A Case Report

**DOI:** 10.7759/cureus.36094

**Published:** 2023-03-13

**Authors:** Alex Gilman, Daniel Von Der Vellen, Ryan Stuart, Russell S Harvey

**Affiliations:** 1 Internal Medicine, Wright State University Boonshoft School of Medicine, Dayton, USA; 2 Internal Medicine, Kettering Medical Center, Kettering, USA

**Keywords:** clinical neuroscience, medication side-effects, visual and auditory hallucinations, cognitive neuroscience, pharmacology, geriatrics and internal medicine

## Abstract

Nitrofurantoin has been utilized for the prevention and treatment of urinary tract infections (UTIs) since the 1950s, and it has been prescribed with increasing frequency since being recommended as a first-line therapy. The adverse neurological and psychiatric effects of antibiotic medications have been well-established. There is evidence to suggest a direct association between acute psychosis and antibiotic exposures. Nitrofurantoin-induced adverse effects have been reported recurrently; however, to the best of our knowledge, a combination of auditory and visual hallucinations with normal baseline mentation and cognition in an immunocompetent geriatric patient, without previously reported hallucinations, have not been reported in the literature so far. We present a case of an 86-year-old Caucasian female who was admitted with audio and visual hallucinations on the fifth day of starting nitrofurantoin therapy for UTI. During her stay, after ruling out all other probable etiologies, it was determined that the likely pathogenesis of the patient’s neuropsychiatric effects was the use of nitrofurantoin.

## Introduction

Urinary tract infections (UTIs), the most common bacterial infections worldwide, are primarily seen in women, with about 50% of women experiencing at least one incidence of UTI in their lifetime [1,2]. Its symptoms often include urgency, frequency, hesitancy, pelvic pain, and dysuria, and it can lead to complicated infections such as pyelonephritis and bacteremia. According to a study from Finland, nearly half of the women who experience one UTI develop recurrent UTIs (RUTI) with 24% having a recurrence within the first six months [3,4]. As women age, there is an increased propensity to develop RUTI due to changes in the hormonal milieu, lifelong exposure to bacteria possibly augmented by sexual activity, and changes in bowel flora [3]. UTIs are the most common infections in patients with diabetes mellitus (DM) [5]. An epidemiological study showed an adjusted incidence for UTI per 1000 person-years in women with type 2 DM of 102.9 (95% CI: 100.5-105.4) versus 76.2 (95% CI: 74.2-78.2) in patients without DM [6]. There is scarce research on the optimal treatment duration of UTI in DM patients. Urogynecological guidelines suggest that patients with well-controlled diabetes may be considered to have an uncomplicated UTI [7]. 

Nitrofurantoin has been available as an antibiotic since the 1950s, and it obtained FDA approval in 1989. Its predominant use is in both the treatment and prophylaxis of uncomplicated UTIs. Even though it has been prescribed and used for decades, its mechanism of action is not fully understood. It is thought to inhibit the bacterial enzymatic synthesis of nucleic acid and cellular structures [8]. Like many antibiotics, nitrofurantoin is known to cause gastrointestinal (GI) upset as an adverse reaction. It has also been documented to cause pulmonary toxicity, liver injury, aplastic anemia, peripheral neuropathy, and hemolytic anemia as rarer adverse reactions [9]. While UTI-specific antibiotics have been implicated in altered mental status and hallucinations, there is scarce data in the literature with regard to nitrofurantoin-specific neuropsychiatric effects as an adverse effect of therapy [10-11].

## Case presentation

An 86-year-old Caucasian female with a pertinent past medical history of paroxysmal atrial fibrillation, insulin-dependent type II DM, diastolic congestive heart failure, recurrent UTIs, hypertension, hypothyroidism, and iron deficiency anemia was admitted with concerns of auditory and visual hallucinations that she had experienced on the fourth and fifth days of starting nitrofurantoin therapy. The patient reported seeing various people whom she did not know in her house interacting among themselves but not with her. The next day, she experienced a vision in which she saw a person with a knife coming toward her. She had also heard music playing in her house a few days prior to admission. Per the patient’s daughter, apart from her hallucinations, the patient had no abnormalities in her baseline cognitive function and exhibited appropriate mentation for age and person on admittance.

Upon being admitted, nitrofurantoin was discontinued due to concerns about it causing encephalopathy. The patient’s vitals were within normal limits, and her only lab abnormalities included hyperglycemia (142 mg/dL) and mild anemia (10.4 g/dL). The patient's home medications included apixaban, duloxetine, lisinopril, levothyroxine, and a short course of nitrofurantoin. The patient had a bedside neurological exam, including cranial nerves, mental status, as well as alertness and orientation. The exam was unremarkable. The patient was alert and oriented to person, time, and place with no other signs of delirium sans the visual hallucinations. In general, the patient was well-kempt and in no general distress with a normocephalic and atraumatic head. The patient wore lipstick, had a full cognitive, pleasant conversation, and had positive hyperacuity of her environment. The remainder of her exam did not reveal any acute findings. 

During the examination, she reported having hallucinations in the room, and she described these as vivid and detailed. She described a door, visible only to her, next to the clothing chest, through which various people entered from the roof. She indicated having little interaction with the visitors. She described seeing children visiting as well. The accuracy of her descriptions was consistent throughout the duration of her hospital stay. The patient indicated that she understood that the hallucinations were not real and she was able to differentiate her hallucinations from reality. Her awareness of this persisted throughout her hospital stay without change or medical staff reassurance.

**Figure 1:**
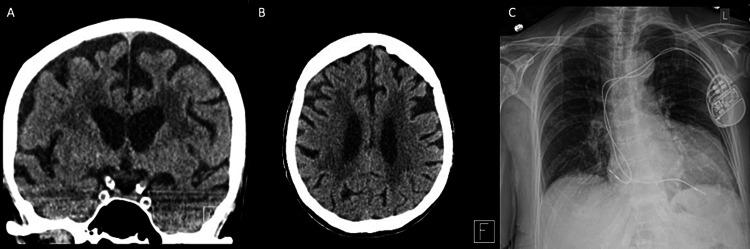
Radiographic imaging CT of the head without contrast in (A) coronal and (B) axial views. (C) AP chest X-ray. Imaging findings were unremarkable for any acute changes that would explain the patient’s hallucinations AP: anteroposterior; CT: computed tomography

An MRI would have been the best imaging modality for identifying structural brain abnormalities; however, it was contraindicated due to a noncompatible pacemaker. A head CT without contrast indicated advanced chronic small vessel ischemia and diffuse cortical atrophy; however, no acute findings or exacerbations that would explain the patient's symptoms were found (Figure 1). The small vessel ischemia and cortical atrophy were not considered causative of her acute hallucination given the onset. Neurologic and psychiatric consults were conducted but reported no abnormal findings to account for an acute or chronic neurologic or psychiatric etiology contributing to the patient’s presentation. The patient was prepped for discharge on the second day of her stay. She was instructed to follow up if the symptoms persisted. On outpatient follow-up, her hallucinations persisted for two days as described, and resolved on the third day following discharge. Given the resolution of symptoms, exclusion of metabolic disturbances, and the low likelihood of late-onset psychiatric disorders, it is likely that the patient’s audiovisual hallucinations were adverse effects of her nitrofurantoin use.

## Discussion

Nitrofurantoin is a commonly prescribed antibiotic for treating UTIs. Though the mechanism of nitrofurantoin is incompletely understood, it is commonly accepted that the medication works through the action of inhibiting various enzymes involved in the synthesis of DNA, RNA, and proteins [8]. It is widely considered to be a safe medication, with relatively few common side effects. Most often, patients may experience nausea, vomiting, and diarrhea; however, more severe side effects such as pulmonary fibrosis, hepatitis and hepatic necrosis, and aplastic anemia have been reported in rare cases [8,9]. Hallucinations are an extremely rare side effect of nitrofurantoin [11]. Other antibiotics have been implicated in causing hallucinations, including penicillin class, trimethoprim-sulfamethoxazole (TMP-SMX), and fluoroquinolones; however, nitrofurantoin as a cause is rarely mentioned in the current literature [10]. A literature review was conducted through PubMed using the search terms such as "hallucination", "auditory", "visual", and "nitrofurantoin". While we found reports of auditory hallucinations as a rare adverse effect of nitrofurantoin usage, no record of a combination of auditory and visual hallucinations was found. A case study from 2014 reported the presentation of auditory hallucinations in a patient with already-established Alzheimer’s disease [11]. Our patient had no history of any memory disorder and no issues related to mentation or cognitive function.

## Conclusions

This report described a unique case of nitrofurantoin-induced audiovisual hallucinations. Our case illustrates that while it remains a diagnosis of exclusion, older patients with comorbidities such as diabetes and frequent UTIs can present with the symptoms described here as an adverse effect of antibiotic use. Various studies have associated hallucinations with antibiotic use. With the increasing use of nitrofurantoin for uncomplicated UTIs and RUTIs, it is pertinent to assess its potential complications in various patient populations.
